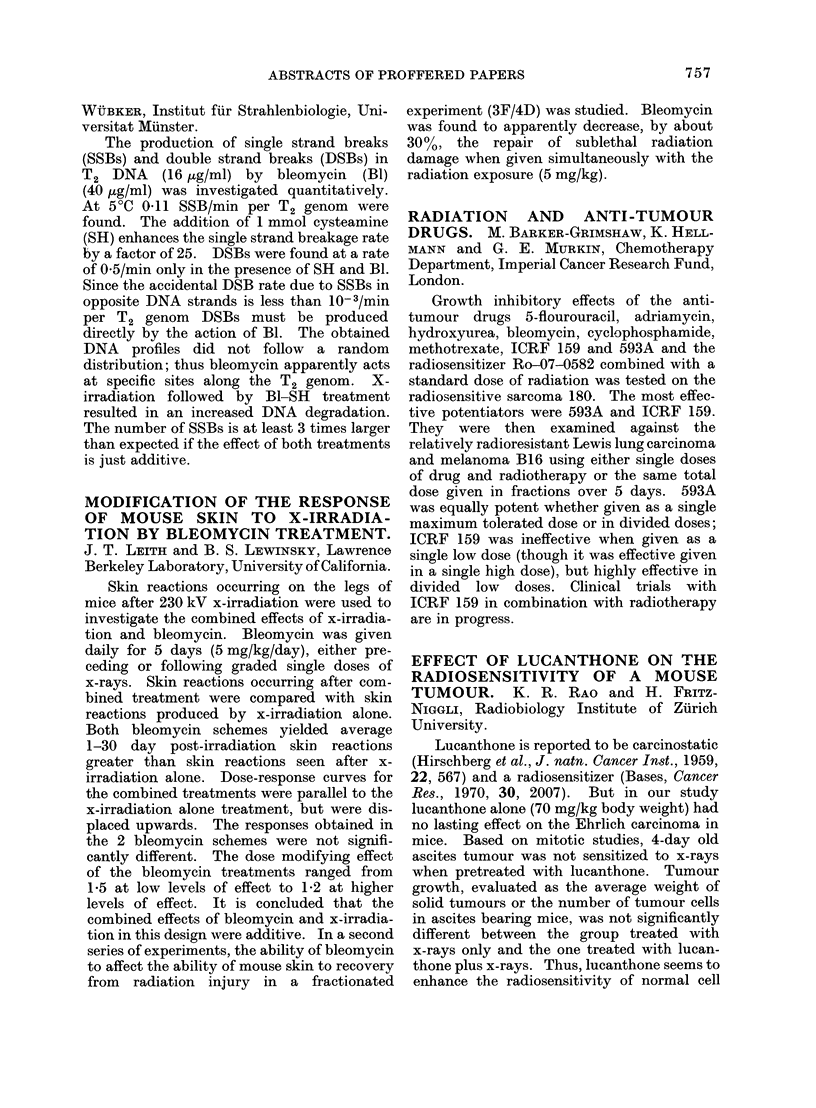# Proceedings: Radiation and anti-tumour drugs.

**DOI:** 10.1038/bjc.1975.309

**Published:** 1975-12

**Authors:** M. Barker-Grimshaw, K. Hellmann, G. E. Murkin


**RADIATION AND ANTI-TUMOUR DRUGS.** M. BARKER-GRIMSHAW, K. HELLMANN and G. E. MURKIN, Chemotherapy Department, Imperial Cancer Research Fund, London.

Growth inhibitory effects of the antitumour drugs 5-flourouracil, adriamycin, hydroxyurea, bleomycin, cyclophosphamide, methotrexate, ICRF 159 and 593A and the radiosensitizer Ro–07–0582 combined with a standard dose of radiation was tested on the radiosensitive sarcoma 180. The most effective potentiators were 593A and ICRF 159. They were then examined against the relatively radioresistant Lewis lung carcinoma and melanoma B16 using either single doses of drug and radiotherapy or the same total dose given in fractions over 5 days. 593A was equally potent whether given as a single maximum tolerated dose or in divided doses; ICRF 159 was ineffective when given as a single low dose (though it was effective given in a single high dose), but highly effective in divided low doses. Clinical trials with ICRF 159 in combination with radiotherapy are in progress.